# Neuromuscular adjustments to unweighted running: the increase in hamstring activity is sensitive to trait anxiety

**DOI:** 10.3389/fphys.2023.1212198

**Published:** 2023-06-02

**Authors:** Camille Fazzari, Robin Macchi, Camélia Ressam, Yoko Kunimasa, Caroline Nicol, Cécile Martha, Benoît Bolmont, Patrick Sainton, Arnaud Hays, Fabrice Vercruyssen, Thomas Lapole, Martin Bossard, Rémy Casanova, Lionel Bringoux, Pascale Chavet

**Affiliations:** ^1^ Aix-Marseille Université, CNRS, ISM, Marseille, France; ^2^ French Institute of Sport (INSEP), Laboratory Sport, Expertise and Performance (EA 7370), Paris, France; ^3^ École Centrale Marseille, Marseille, France; ^4^ Department of Health and Sport Sciences, Niigata University, Niigata, Japan; ^5^ Université de Lorraine, 2LPN-CEMA Group, Metz, France; ^6^ Université de Toulon, IAPS, Toulon, France; ^7^ Université Jean Monnet, Université Savoie Mont-Blanc, LIBM, St-Etienne, France; ^8^ Université Gustave Eiffel, COSYS-PICS-L, F-77454 Marne-la-Vallée, France

**Keywords:** neuromuscular adjustments, unweighting, reloading, lower body positive pressure, running, repeatability, trait anxiety

## Abstract

**Introduction:** Originally developed for astronauts, lower body positive pressure treadmills (LBPPTs) are increasingly being used in sports and clinical settings because they allow for unweighted running. However, the neuromuscular adjustments to unweighted running remain understudied. They would be limited for certain lower limb muscles and interindividually variable. This study investigated whether this might be related to familiarization and/or trait anxiety.

**Methods:** Forty healthy male runners were divided into two equal groups with contrasting levels of trait anxiety (high, ANX^+^, *n* = 20 vs. low, ANX^−^, *n* = 20). They completed two 9-min runs on a LBPPT. Each included three consecutive 3-min conditions performed at 100%, 60% (unweighted running), and 100% body weight. Normal ground reaction force and electromyographic activity of 11 ipsilateral lower limb muscles were analyzed for the last 30 s of each condition in both runs.

**Results:** Unweighted running showed muscle- and stretch-shortening cycle phase-dependent neuromuscular adjustments that were repeatable across both runs. Importantly, hamstring (BF, biceps femoris; STSM, semitendinosus/semimembranosus) muscle activity increased during the braking (BF: +44 ± 18%, *p* < 0.001) and push-off (BF: +49 ± 12% and STSM: +123 ± 14%, *p* < 0.001 for both) phases, and even more so for ANX^+^ than for ANX^−^. During the braking phase, only ANX^+^ showed significant increases in BF (+41 ± 15%, *p* < 0.001) and STSM (+53 ± 27%, *p* < 0.001) activities. During the push-off phase, ANX^+^ showed a more than twofold increase in STSM activity compared to ANX^−^ (+119 ± 10% vs. +48 ± 27, *p* < 0.001 for both).

**Conclusion:** The increase in hamstring activity during the braking and push-off phases may have accelerated the subsequent swing of the free-leg, likely counteracting the unweighting-induced slowing of stride frequency. This was even more pronounced in ANX^+^ than in ANX^−^, in an increased attempt not to deviate from their preferred running pattern. These results highlight the importance of individualizing LBPPT training and rehabilitation protocols, with particular attention to individuals with weak or injured hamstrings.

## 1 Introduction

Human locomotion has been studied in actual hypogravity (i.e., during space and parabolic flights) and simulated hypogravity (i.e., on Earth) (for a review, see Lacquaniti et al., 2017). Among the most commonly used simulators, lower body positive pressure treadmills (LBPPTs) apply a lifting force at approximately the runner’s center of mass through small increases in air pressure within their airtight chamber, providing vertical body weight (BW) support (Whalen and Hargens, 1992). Originally developed for astronauts, LBPPTs are now being used in sports and clinical settings. Therefore, a better understanding of the biomechanical and neuromuscular adjustments to unweighted and reloaded running may contribute to the success of both space missions and terrestrial training and rehabilitation protocols.

The biomechanical adjustments to unweighted running on a LBPPT have been relatively well studied. In particular, it has been shown that unweighted running reduces the forces acting on the musculoskeletal system, as evidenced by the reduced active peak force. It also induces a slowing of stride frequency, due to a prolonged flight time associated with a slightly shortened contact time (Grabowski and Kram, 2008; Sainton et al., 2015; Sainton et al., 2016). Reloaded running shows opposite biomechanical adjustments, albeit with some minor aftereffects. Specifically, Sainton et al. (2015) reported the persistence of a slower stride frequency, attributed to a longer flight time, up to 3 min after returning to 100% BW.

Paradoxically, only six studies have investigated the underlying neuromuscular adjustments to unweighted running on a LBPPT (for a review, see Farina et al., 2017). All agreed that lower limb muscle activity decreases with BW support. However, the first study reported a preservation of the muscle activity pattern (Liebenberg et al., 2011), while later studies have instead highlighted its complex reorganization. They revealed that the neuromuscular adjustments to unweighted running were dependent on the muscle and the phase of the running cycle phase (i.e., stance and flight phases). Importantly, they showed no change in the activity of the medial (semitendinosus) and lateral (biceps femoris) hamstring muscles during the stance phase (Mercer et al., 2013; Hunter et al., 2014; Jensen et al., 2016). When differentiating the stretch-shortening cycle (SSC) phases, Sainton et al. (2016) reported unchanged muscle activity during the preactivation phase, but decreased vasti and triceps surae activity during the braking and push-off phases. They also found opposite neuromuscular adjustments upon return to 100% BW. However, they only monitored vasti and triceps surae activity in a limited cohort of 9 runners. As a result, it is not clear whether such SSC phase-dependent neuromuscular adjustments could also apply to other lower limb muscles, particularly the hamstring muscles.

Further emphasizing the complex reorganization of the muscle activity pattern, the neuromuscular adjustments to unweighted running appear to be nonlinear with BW reduction. The decrease in lower limb muscle activity is reported to be less than the decrease in BW. Furthermore, it would be extremely limited below 30% BW, suggesting a “ceiling effect’ in the reduction of muscle activity (Liebenberg et al., 2011; Mercer et al., 2013). It is worth noting that in the aforementioned studies, the participants were only familiarized with running on a LBPPT at 100% BW. Thus, the observed “ceiling effect” could be due to a lack of familiarization to unweighted running. In line with this suggestion, Hunter et al. (2014) suggested that with repeated and/or prolonged training on a LBBPT, runners could progressively modify their running pattern, to the point where hamstring muscle activity could be reduced. Similarly, Naylon et al. (2022) recently reported that vastus medialis and gastrocnemius medialis activities progressively decreased during the first 20 min of unweighted running (at 70% BW). It is therefore of interest to investigate the repeatability of the neuromuscular adjustments between a first and a second unweighted run.

Finally, the neuromuscular adjustments to unweighted running show a high inter-individual variability. In addition, some runners expressed discomfort with the slowing of their stride frequency at 60% BW (Sainton et al., 2015). Although the reported inter-individual variability is likely to be multifactorial, it could be partly explained by psychological characteristics, such as state or trait anxiety. Whereas state anxiety is a transient emotion, trait anxiety is a predisposition to perceive a wide range of situations as threatening (Spielberger et al., 1983). State anxiety is known to affect balance and locomotion. In particular, muscle co-contraction has been found to be greater when state anxiety increases (Carpenter et al., 1999; Carpenter et al., 2001). It has also been shown that stride frequency is higher and stride length is shorter, resembling a “more conservative gait pattern” (McKenzie and Brown, 2004; Brown et al., 2006; Nibbeling et al., 2012). However, it remains unclear whether trait anxiety also affects the strategies used to maintain sensory-motor performance. One of the few available studies reported that balance is differentially altered in an anxiogenic situation depending on the level of trait anxiety (Hainaut et al., 2011). It is therefore relevant to examine whether the neuromuscular adjustments to unweighted running might also be influenced by trait anxiety.

In this context, the present study was designed to further our understanding of the neuromuscular adjustments to unweighted and reloaded running, and the extent to which they may be influenced by familiarization and trait anxiety. Firstly, it was hypothesized that unweighted and reloaded running would result in muscle- and SSC phase-dependent neuromuscular adjustments. Secondly, it was expected that a second unweighted run would show more pronounced neuromuscular adjustments, i.e., a greater decrease in muscle activity due to familiarization. Finally, it was expected that individuals with high levels of trait anxiety would show a more conservative running pattern compared to those with low levels of trait anxiety, i.e., limited neuromuscular adjustments to unweighted running.

## 2 Materials and methods

### 2.1 Participants

Participants were selected from a cohort of 228 male students (age 19 ± 1 years old). All of them had not suffered a musculoskeletal injury for more than 1 year, and had no history of neurological impairment. Their trait anxiety was assessed using the French version of the State-Trait Anxiety Inventory (STAI Y2-form, Spielberger et al., 1983). From this cohort, two groups were then constituted by selecting the participants whose trait anxiety was at the extremes of the distribution. According to the experimental design, for a medium effect size (ƞ^2^ = 0.06), at least 14 participants were needed in each group to obtain a statistical power of 80%. We decided to include 20 men in each group: those with lower trait anxiety formed the low trait anxiety group (ANX^−^: score ≤28, age 19 ± 1 years old, height 178 ± 7 cm, weight 71.6 ± 8.0 kg) and those with higher trait anxiety formed the high trait anxiety group (ANX^+^: score ≥43, age 19 ± 2 years old, height 176 ± 6 cm, weight 66.9 ± 7.6 kg). This double-blind study was approved by the National Ethics Committee for Research in Sports Sciences (reference number: CERSTAPS IRB00012476-2021-31-03-96). In accordance with the Declaration of Helsinki, participants gave written informed consent before the experiment.

### 2.2 Experimental design

**FIGURE 1:**
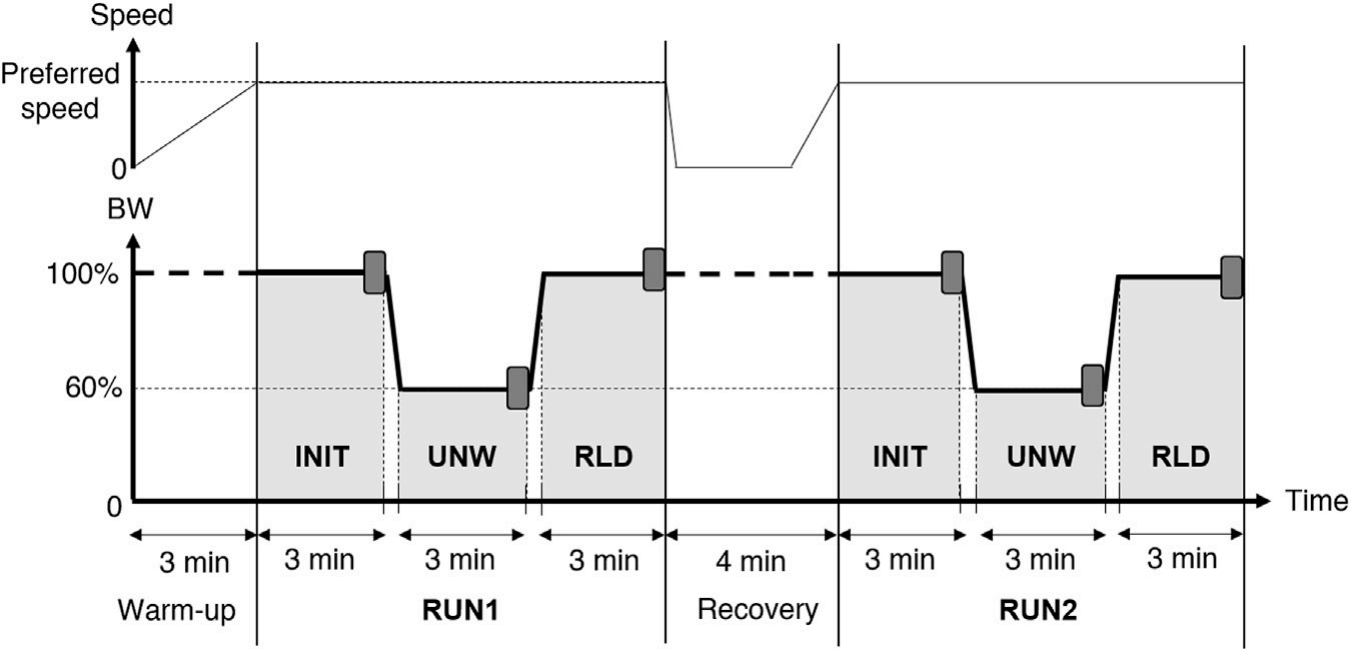
Experimental protocol. The LBPPT allowed running at either 100% or 60% body weight (BW). The darker rectangles indicate the 30 s analyzed at the end of the initial (INIT), unweighted (UNW), and reloaded (RLD) conditions in both runs (RUN1 and RUN2).

Participants ran on a LBPPT (VIA400X AlterG^®^, Fremont, CA, United States) at their preferred speed (ANX^+^: 3.0 ± 0.2 m.s^−1^; ANX^−^: 3.0 ± 0.2 m.s^−1^). The latter was self-selected during a 100% BW familiarization run performed during the week prior to the experimental protocol. The experimental protocol began with a 3-min LBPPT running warm-up to gradually reach the preferred speed. It then consisted of two 9-min runs (RUN1 and RUN2) separated by a 4-min recovery period (3 min standing and 1 min walking). Each run consisted of 3 consecutive 3-min conditions. The initial (INIT) and reloaded (RLD) conditions were performed at 100% BW, whereas the unweighted (UNW) intermediate condition was performed at 60% BW. The transition phases between the conditions lasted 12 ± 2 s, during which BW was linearly decreased (from INIT to UNW) or increased (from UNW to RLD) (Figure 1).

### 2.3 Data recordings

The normal component of the ground reaction force was recorded using instrumented insoles (Loadsol, Novel^®^, Munich, Germany; 100 Hz) placed in standardized running shoes (Run active, Kalenji^®^). Triaxial acceleration at the pelvic level and the right forefoot as well as surface electromyographic (EMG) activity were recorded using bipolar electrodes (miniWave COMETA^®^, Milan, Italy; 2000 Hz). They were fixed on the skin over 11 muscles of the right lower limb, including gluteus maximus (GM), vastus medialis (VM), vastus lateralis (VL), rectus femoris (RF), semitendinosus/semimembranosus (STSM), biceps femoris (BF), soleus (SOL), gastrocnemius medialis (GaM), gastrocnemius lateralis (GaL), tibialis anterior (TA) and peroneus longus (PL). Skin preparation and positioning of the bipolar electrodes followed the SENIAM recommendations (Hermens et al., 2000). Heart rate (RS 800 Polar^®^, Kempele, Finland) and rate of perceived exertion expressed on a scale of 6–20 (Borg, 1970) were also recorded. State anxiety was assessed before and after RUN1 (STAI Y1-form, Spielberger et al., 1983). In the latter case, the questionnaire was completed on the basis of the participants’ general feelings during RUN1.

### 2.4 Data analysis

All data analyses were performed on the last 30 s of each condition. Touchdown and toe-off were identified from the normal component of the ground reaction force using a threshold set at 50 N. Acceleration signals were low-pass filtered using a 4th order Butterworth zero-phase filter with a 10 Hz cut-off frequency. The vertical acceleration recorded at the pelvic level was double integrated to identify the end of the braking phase, corresponding to the minimum vertical position of the center of mass. The flight, contact, stride, braking and push-off times were then calculated, as well as the pelvis vertical displacement during the braking and the push-off phases. Active peak force, and mean vertical force during the braking and push-off phases were also computed. The foot strike pattern (rearfoot or midfoot/forefoot) was obtained from the sensors located in the anterior and posterior areas of the instrumented insoles.

The recorded EMG signals were band-pass filtered (10-500 Hz), rectified and low pass filtered using a 75 Hz critically damped filter (Morio et al., 2012). After subtracting the minimum value, the amplitude of each EMG recording was normalized to its maximum activity recorded during INIT of RUN1. The mean activity of each recorded muscle was calculated from a root mean square analysis for the preactivation, braking and push-off phases. The preactivation phase was defined as the 100 milliseconds before touchdown (Komi et al., 1987).

### 2.5 Statistics

For each variable, a linear mixed model [R package LmerTest, Kuznetsova et al., 2017)] was performed using restricted maximum likelihood estimation. Condition (INIT, UNW, RLD), Run (RUN1, RUN2) and Group (ANX^−^, ANX^+^) were considered as fixed effects when appropriate, and preferred running speed was considered as covariate. The intercepts for the participants and the slope per condition depending on the foot strike pattern were chosen as random effects. The significance of the random effects was tested. The number of fixed effects was chosen by likelihood ratio tests of model comparisons using a backward selection method. Analysis of variance (degrees of freedom estimated using the Satterthwaite formula) was performed on the selected model. This was followed by pairwise comparisons with Tukey’s adjustment (for main effects) and Holm’s adjustment (for interaction effects). If the normality of the residuals was violated (Shapiro-Wilk test), a permutation analysis of variance of the linear mixed model was performed (R package lmPerm, number of permutations: 10,000). The effect size (ES) was calculated using Cohen’s d coefficient (Cohen, 1988) and assessed using the following thresholds: 0.2 to <0.6, 0.6 to <1.2 and greater than 1.2 for small, moderate and large effects, respectively (Hopkins et al., 2009). Trivial effects (ES < 0.2) are not reported. All the significance levels were set at α < 0.05 and the statistical analyses were performed using a custom R script (v3.6.3, R Foundation for Statistical Computing, Vienna, Austria). All results are presented as estimated mean ± standard error.

## 3 Results

### 3.1 Unweighting and reloading adjustments

**FIGURE 2:**
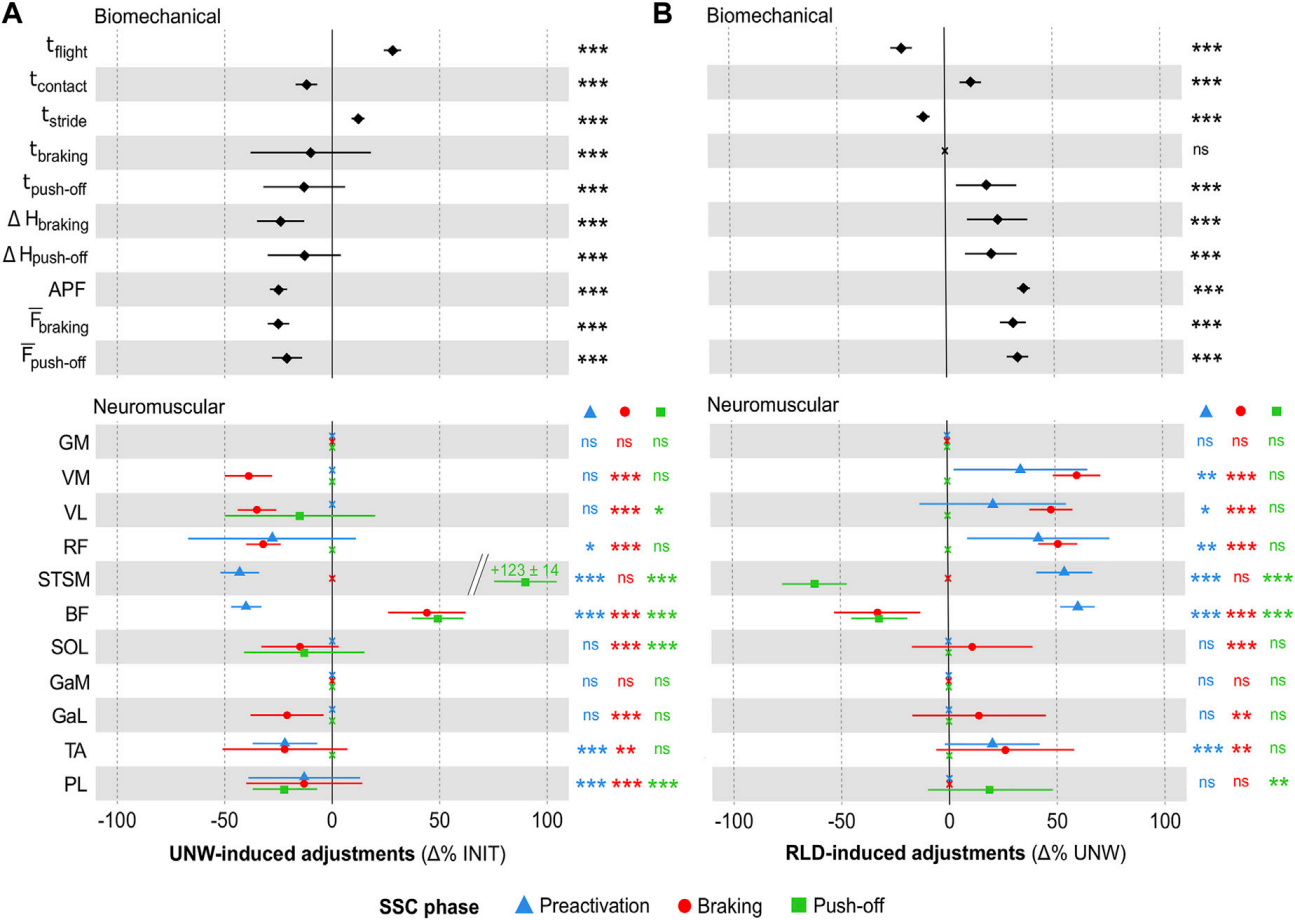
Unweighting and reloading adjustments. **(A)** Mean (± standard error) UNW-induced adjustments (in Δ% as compared to INIT) in the biomechanical and neuromuscular variables (preactivation indicated by blue triangles, braking by red circles, and push-off by green squares). **(B)** Mean (± standard error) RLD-induced adjustments (in Δ% as compared to UNW) in the biomechanical and neuromuscular variables. For both panels, significant adjustments (as compared to INIT and UNW, respectively) are shown as follows: ***p < 0.001, **p < 0.01, *p < 0.05. Non-significant (ns) adjustments are displayed as crosses. INIT, UNW and RLD for initial, unweighted and reloaded conditions; SSC, stretch-shortening cycle; t_flight_, flight time; t_contact_, contact time; t_stride_, stride time; t_braking_, braking time; t_push-off_, push-off time; ΔH_braking_ and ΔH_push-off_ for pelvic vertical displacement during the braking and push-off phases; APF, active peak force; ${\bar{F}}_{braking}$ and ${\bar{F}}_{push-off}$ and for mean vertical force during the braking and push-off phases; GM, gluteus maximus; VM, vastus medialis; VL, vastus lateralis; RF, rectus femoris; STSM, semitendinosus/semimembranosus; BF, biceps femoris; SOL, soleus; GaM, gastrocnemius medialis; GaL, gastrocnemius lateralis; TA, tibialis anterior; PL, peroneus longus.

The biomechanical and neuromuscular adjustments to unweighting (UNW vs. INIT) are shown in Figure 2A (see Supplementary Figure S1A for individual unweighting adjustments). The biomechanical analysis (Figure 2A top panel) showed longer flight (+28 ± 4%, ES = 2.9) and stride (+12% ± 3%, ES = 2.3) times, shorter contact (−2 ± 5%, ES = 1.3), braking (−10% ± 28%, ES = 0.9) and push-off (−13% ± 19%, ES = 0.5) times. It revealed smaller pelvis vertical displacement during braking (−24% ± 11%, ES = 1.0) and push-off (−13% ± 17%, ES = 0.6) phases. It also showed lower active peak force (−25% ± 4%, ES = 2.0), and mean braking (−25% ± 5%, ES = 1.7) and push-off (−21% ± 7%, ES = 1.2) forces. The EMG analysis revealed muscle- and SSC phase-dependent neuromuscular adjustments (Figure 2A bottom panel). Preactivation was lower for some thigh (RF: ES = 0.3, STSM: ES = 1.6 and BF: ES = 1.2) and shank (TA: ES = 0.5 and PL: ES = 0.2) muscles. The braking phase showed an overall decrease in muscle activity for the quadriceps muscle group (VM: ES = 1.8, VL: ES = 1.7 and RF: ES = 0.8) and for most of the shank muscles (SOL: ES = 0.6, GaL: ES = 0.7, TA: ES = 0.4 and PL: ES = 0.4), except for GaM. Only the hamstring muscles showed either an increased (BF: ES = 0.4) or an unchanged (STSM) activity. The push-off phase showed lower activities for some muscles (VL: ES = 0.3, SOL: ES = 0.4 and PL: ES = 0.6), but higher activities for the hamstring muscles (STSM: ES = 1.0 and BF: ES = 0.8). UNW was also associated with lower mean heart rate (151 ± 2 bpm vs. 167 ± 2 bpm; −9 ± 6%, ES = 1.1, *p* < 0.001) and rate of perceived exertion (9.0% ± 0.3% vs. 11.0 ± 0.3; −18% ± 8%, ES = 1.2, *p* < 0.001).

The biomechanical and neuromuscular adjustments to reloading (RLD vs. UNW) are shown in Figure 2B (see Supplementary Figure S1B for individual reloading adjustments). They were opposite to those induced by UNW, with most of the biomechanical (Figure 2B top panel) and neuromuscular (Figure 2B bottom panel) parameters returning to their initial values within 3 min. However, some aftereffects (RLD vs. INIT) were noted as follows. The biomechanical analysis showed longer flight (+3 ± 37%, *p* < 0.05, ES = 0.4) and stride (+1 ± 28%, *p* < 0.001, ES = 0.4) times, a shorter braking time (−7% ± 40%, *p* < 0.05, ES = 0.4) and a higher mean push-off force (+5 ± 26%, *p* < 0.001, ES = 0.3) in RLD than in INIT. The EMG analysis revealed lower muscle activity in RLD than in INIT. STSM activity was lower during the preactivation phase (−13% ± 30%, *p* < 0.01, ES = 0.3). Shank muscle activity was lower during the braking (GaL and PL: −9 ± 38%, *p* < 0.05, ES = 0.2 for both) and push-off (SOL: −12% ± 29%, *p* < 0.01, ES = 0.4; GaL: −11% ± 41%, *p* < 0.05, ES = 0.3; PL: −10% ± 34%, *p* < 0.01, ES = 0.2) phases. Heart rate and rate of perceived exertion were higher in RLD than in INIT (+2 ± 22%, *p* < 0.001, ES = 0.3 and +11 ± 14%, *p* < 0.001, ES = 0.7 respectively).

### 3.2 Repeatability of unweighting adjustments

The biomechanical and neuromuscular adjustments to UNW were repeatable, except for the following few parameters. Compared to RUN1, RUN2 showed a larger decrease in braking time with UNW (−19% ± 15%, *p* < 0.001, ES = 1.2 vs. −10% ± 28%, *p* < 0.01, ES = 0.6). This was also true for GaL activity during the push-off (−15% ± 25%, *p* < 0.01, ES = 0.5 vs. −10% ± 31%, *p* < 0.001, ES = 0.4) and heart rate (−11% ± 5%, ES = 1.2 vs. −9 ± 6%, ES = 1.0, *p* < 0.001 for both).

Regardless of the condition (INIT, UNW, RLD), the EMG analyses revealed lower muscle activities in RUN2 than in RUN1. STSM activity was lower during the preactivation phase (−12% ± 38%, *p* < 0.01, ES = 0.2), as were VL (−9 ± 37%, *p* < 0.01, ES = 0.2) and most shank muscles activity during the braking phase (SOL: −13% ± 20%, GaL: −16% ± 23% and PL: −13% ± 20%; *p* < 0.001, ES = 0.4 for all three). During the push-off phase, SOL (−11% ± 32%, *p* < 0.01, ES = 0.2) and PL (−17% ± 21%, *p* < 0.001, ES = 0.4) activities were also lower, whereas GM (+41 ± 39%, *p* < 0.05, ES = 0.3) activity was higher.

### 3.3 Influence of trait anxiety on unweighting adjustments

ANX^+^ and ANX^−^ did not differ significantly with respect to age, height, weight and preferred running speed. ANX^+^ showed higher state anxiety than ANX^−^ (33 ± 2 vs. 27 ± 2, *p* < 0.01, ES = 0.8), but UNW did not affect state anxiety (assessed before and after RUN1, *p* = 0.34).

**FIGURE 3:**
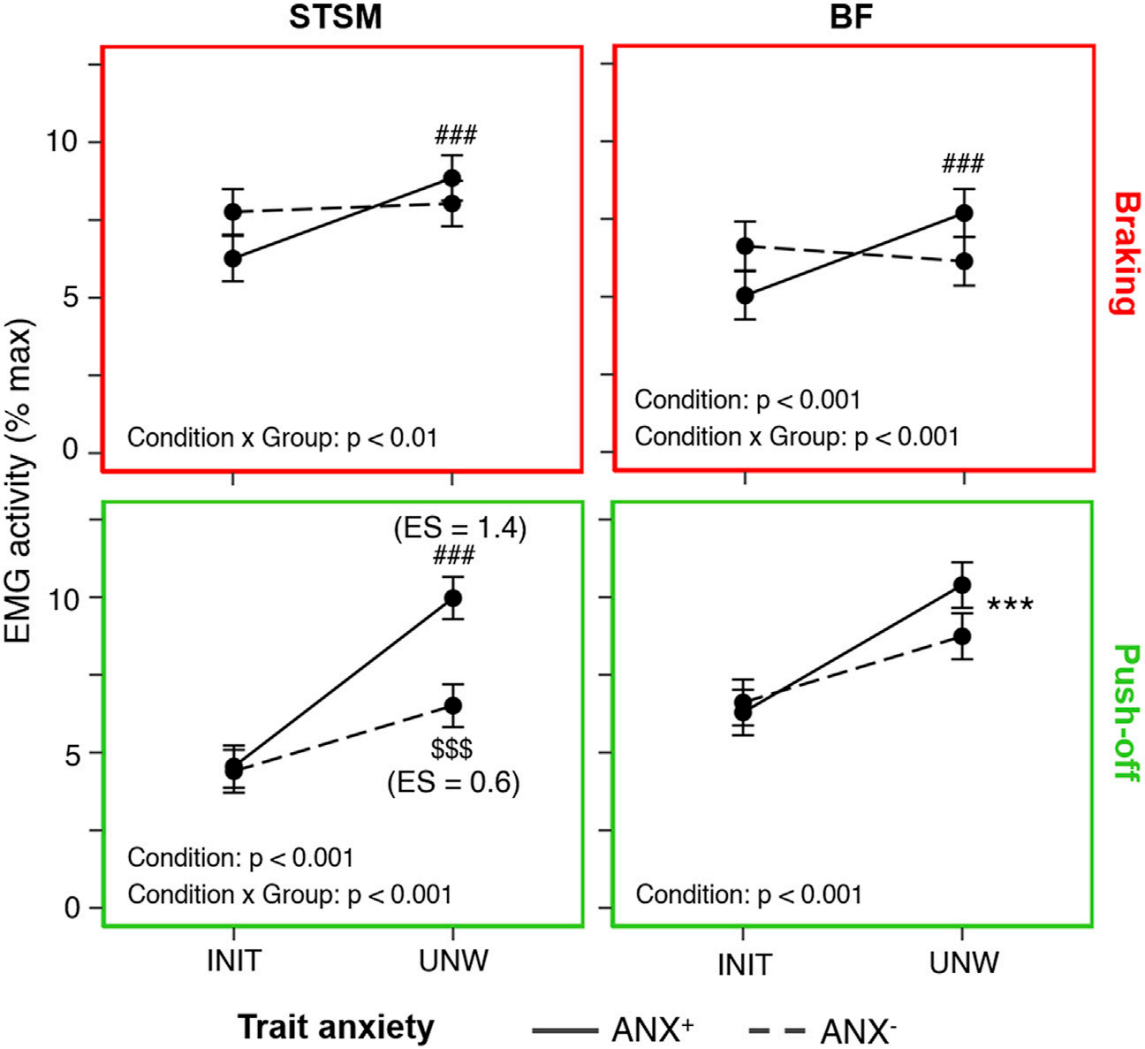
Influence of trait anxiety on unweighting adjustments. Mean (± standard error) hamstring (BF, biceps femoris and STSM, semitendinosus/semimembranosus) activity (in % max) recorded in the initial (INIT) and unweighted (UNW) conditions during the braking (upper panels framed in red) and push-off (lower panels framed in green) phases for the high-trait (ANX+, solid lines) and low-trait (ANX−, dashed lines) anxiety groups. Significant adjustments (as compared to INIT) are shown for the whole group (*), ANX+ (#) and ANX− ($), with the number of symbols indicating the statistical level (three for *p* < 0.001). ES, effect size.

ANX^+^ showed more pronounced adjustments to UNW than ANX^−^. They showed a larger increase in flight time (+28 ± 3%, *p* < 0.001, ES = 4.2 vs. +24 ± 4%, *p* < 0.001, ES = 2.3) and a larger decrease in VM activity during the braking phase (−38% ± 8%, *p* < 0.001, ES = 2.6 vs. −26% ± 12%, *p* < 0.001, ES = 1.3). Importantly, they also showed a larger increase in hamstring muscle activity with UNW. More specifically, only ANX^+^ showed an increase in STSM (+53 ± 27%, *p* < 0.001, ES = 0.8 vs. non-significant) and BF (+41 ± 15%, *p* < 0.001, ES = 0.7 vs. non-significant) activities during the braking phase. Furthermore, ANX^+^ showed a larger increase in STSM activity than ANX^−^ during the push-off phase (+119 ± 10%, *p* < 0.001, ES = 1.4 vs. +48 ± 27%, *p* < 0.001, ES = 0.6) (Figure 3).

## 4 Discussion

This study enriched our understanding of the neuromuscular adjustments previously reported during UNW and RLD running (for a review, see Farina et al., 2017), while demonstrating their repeatability. Importantly, it revealed an increase in hamstring muscle activity during UNW that was sensitive to the level of trait anxiety.

### 4.1 Unweighting and reloading adjustments

Confirming our first hypothesis, UNW and RLD showed muscle- and SSC phase-dependent neuromuscular adjustments. This supports and complements the previous findings of Sainton et al. (2016) by monitoring the activity of more lower limb muscles in a larger cohort of runners.

UNW resulted in a decrease in TA preactivation, consistent with the previously reported shift towards a more forefoot strike (Smoliga et al., 2015; Neal et al., 2016). However, it did not affect the preactivation of most lower limb extensors muscles (GM, vasti and triceps surae). These results suggest the involvement of passive mechanisms as formalized by the mass-spring model when flight time increases (Seyfarth et al., 2003). The elevation of the runner’s center of mass probably favored passive retraction of the swing leg, resulting in a steeper angle of attack at touchdown. This would result in almost constant leg stiffness, even in the absence of increased preactivation (Müller and Blickhan, 2010). The lack of visual feedback on foot touchdown may also explain the unchanged preactivation. Indeed, a “default pattern of motor commands” has been reported during landing movements and during running on uneven ground when vision is not available (Santello et al., 2001; Müller et al., 2014). In the current study, the braking and push-off phases showed a decrease in the activity of most of the lower limb muscles, particularly for the quadriceps (VM, VL, RF) and triceps surae (SOL, GaL, except GaM) muscle groups, and for the PL. The lifting force provided by the LBPPT probably explains the reduced lowering of the center of mass during the braking phase. Therefore, the role of the lower limb extensor muscles in supporting and propelling the BW during stance is reduced, and less muscle activity is required (Ferris et al., 2001). In contrast, the activity of the hamstring muscles (STSM and BF) increased during both the braking and push-off phases. This is consistent with the decrease in peak knee flexion, followed by the increase in knee flexion at toe-off (Neal et al., 2016). Furthermore, previous studies have already reported that their activity remains unchanged, rather than decreases during the stance phase (Mercer et al., 2013; Hunter et al., 2014; Jensen et al., 2016). This increased hamstring activity during the stance phase is thought to accelerate the subsequent swing of the free-leg, likely counteracting the UNW-induced decrease in stride frequency.

These neuromuscular adjustments to UNW running have important implications for the training and rehabilitation of astronauts, athletes, and patients. Indeed, the reduced rate of perceived exertion may give the latter the false impression that all muscles are under less stress. However, the observed increase in hamstring activity should prompt coaches and clinicians to be cautious with astronauts, athletes and patients with weak or injured hamstrings (Hunter et al., 2014; Farina et al., 2017). This is especially important because the participants tend to lean forward on the LBPPT support structure (McNeill et al., 2015). This suggests greater anterior pelvic tilt, which is known to increase the risk of hamstring injury (Hoskins & Pollard, 2005; Schuermans et al., 2017). Therefore, if the increase in hamstring activity were to be confirmed in actual hypogravity, pre-flight training of astronauts may benefit from strengthening this muscle group and/or improving lumbar–pelvic neuromuscular control.

RLD resulted in opposite biomechanical and neuromuscular adjustments to those induced by UNW, with some minor aftereffects. Consistent with Sainton et al. (2015), prolonged flight and stride times persisted up to 3 min after returning to 100% BW. In this line, Brennan et al. (2018) recently reported a moderate (+4.3%) but persistent increase in stride length at 100% BW after 8 weeks of LBPPT (3 times a week for 20 min at 85% BW). Such aftereffects are classically reported in goal-directed arm movements when tested in a normal force field after the adaptation to a modified force field (e.g., Lackner and Dizio, 1994), and suggest that an adaptation of motor commands occurred during UNW. The persistence of reduced shank muscle activity up to 3 min after return to 100% BW was expected to improve running economy. However, both heart rate and rate of perceived exertion were found to be slightly higher. Furthermore, most of the runners reported an uncomfortable feeling of heavy legs after returning to 100% BW (Grabowski and Kram, 2008; Sainton et al., 2015). This supports the aforementioned idea that muscle activity and subjective sensations may be uncorrelated when running on a LBPPT (Farina et al., 2017).

### 4.2 Repeatability of unweighting adjustments

Contrary to our second hypothesis, the second unweighted run (UNW of RUN2) did not show more pronounced neuromuscular adjustments than the first unweighted run (UNW of RUN1). Rather, it highlighted their repeatability. Only GaL muscle activity during the push-off phase showed a greater decrease with UNW in RUN2 than in RUN1. This is usually considered to reflect an optimization of the SSC (Komi and Nicol, 2010), which is confirmed by the greater decreases in braking time and heart rate. Along these lines, McNeill et al. (2015) reported that full metabolic accommodation (i.e., reduction and stabilization of the oxygen consumption) may require four sessions of 15 min when running on a LBPPT at 50, 70% and 90% BW. Similarly, Naylon et al. (2022) showed that VM and GaM activities decreased during the first 20 and 10 min, respectively, before stabilizing when running on a LBPPT at 70% BW.

Interestingly, RUN2 showed lower muscle activity than RUN1, regardless of the condition. This suggests that neuromuscular habituation to LBPPT running occurred between RUN1 and RUN2. Even on a classical treadmill, kinematic habituation (stabilization of stride length and stride frequency) is known to occur after three 15-min running sessions (Schieb, 1986).

### 4.3 Influence of trait anxiety on unweighting adjustments

Contrary to our third hypothesis, individuals with high levels of trait anxiety (ANX^+^) showed more pronounced rather than limited adjustments. As a preamble, ANX^+^ showed a higher state anxiety than ANX^−^, which is consistent with the high correlation classically reported between state and trait anxiety (Spielberger et al., 1983). Nevertheless, UNW was not stressful enough to induce an increase in runners’ state anxiety.

Despite the unchanged state anxiety, trait anxiety influenced the neuromuscular adjustments to UNW. During the braking phase, ANX^+^ showed an increase in hamstring muscle activity (STSM and BF), whereas ANX^−^ did not. During the subsequent push-off phase, they showed a greater increase in STSM activity than the ANX^−^. These results may provide an explanation for the large inter-individual variability previously reported in hamstring muscle activity during unweighted walking (Ivanenko et al., 2002; Lacquaniti et al., 2017). Notably, this further increase in hamstring muscle activity was not associated with an increase in lower limb stiffness (i.e., in muscle co-contraction). In fact, ANX^+^ showed a concomitant greater decrease in VL activity during the braking phase. The latter is attributed to reciprocal inhibition, which prevents functional antagonist muscles from working against each other (Jankowska et al., 1965). The earlier and greater increase in hamstring muscle activity shown by ANX^+^ is considered to be an increased attempt not to deviate from their preferred running pattern, and thus from their preferred stride frequency. In support of this suggestion, previous studies have shown that participants exhibit a “more conservative gait pattern,” characterized by a higher stride frequency, when state anxiety increases (McKenzie and Brown, 2004; Brown et al., 2006; Nibbeling et al., 2012). Such a strategy might also be adopted when trait anxiety is higher. It might have been more pronounced, or even different, if UNW running had actually been experienced as stressful. It should be noted that the UNW-induced decrease in stride frequency did not differ between ANX^+^ and ANX^−^. Thus, the specific neuromuscular adjustments observed in ANX^+^ were not sufficient to effectively counteract its slowing. Yet, they may explain the greater increase in flight time seen in ANX^+^ than in ANX^−^, as hamstring muscle activity contributes to vertical force production.

These findings suggest that not all individuals adjust equally to unweighted running as a function of personality traits. Importantly, the aforementioned risk of hamstring injury may be greater in ANX^+^ than in ANX^−^. This has important implications for the success of future space missions, which are likely to involve more heterogeneous astronaut crews. It should also be considered for the success of training and rehabilitation protocols, where the heterogeneity of athletes and patients is even more pronounced.

### 4.4 Limits and perspectives

Given the sex differences reported in endurance running (Besson et al., 2022) and in the biomechanical adjustments to UNW running (Gojanovic et al., 2012), the inclusion of only males limits the generalizability of the findings. Therefore, further studies should investigate the neuromuscular adjustments to UNW running in women. Furthermore, measurements of joint kinematics are lacking to refine our understanding of the functional role of the hamstring muscles during the stance phase. Finally, further studies are needed to verify whether the neuromuscular adjustments can be improved by more and/or longer UNW runs.

## 5 Conclusion

The present study refined our knowledge of the biomechanical and neuromuscular adjustments to UNW and RLD running, and demonstrated their repeatability. The current recording of a large number of lower limb muscle activities confirmed that the neuromuscular adjustments are muscle- and SSC phase-dependent. Importantly, UNW showed an increase, rather than a decrease, in hamstring muscle activity during the braking and push-off phases. This adjustment may have accelerated the subsequent swing of the free-leg, likely counteracting the UNW-induced slowing of stride frequency. It was even more pronounced in ANX^+^ than in ANX^−^, in an increased attempt not to deviate from their preferred running pattern. These results highlight the importance of individualizing LBPPT training and rehabilitation protocols, with particular attention to individuals with weak or injured hamstrings.

## Data Availability

The raw data supporting the conclusion of this article will be made available by the authors, without undue reservation.
