# Supplementary material for: Neuromuscular adjustments to unweighted running: the increase in hamstring activity is sensitive to trait anxiety
Source: Front Physiol. 2023 Jun 2;14:1212198. doi: 10.3389/fphys.2023.1212198 (PMC10272775; doi:10.3389/fphys.2023.1212198)
Supplement: Supplementary file 1 [file DataSheet1.pdf]

Supplementary Figure S1. Unweighting and reloading adjustments. (A) Individual UNW-induced adjustments (in Δ% as compared to INIT) in the biomechanical and neuromuscular variables (preactivation indicated by blue triangles, braking by red circles, and push-off by green squares). **(B)** Individual RLD-induced adjustments (in Δ% as compared to UNW) in the biomechanical and neuromuscular variables. For both panels, significant adjustments (as compared to INIT and UNW, respectively) are shown as follows: ***p < 0.001, **p < 0.01, *p < 0.05. Non-significant (ns) adjustments are displayed as crosses. INIT, UNW and RLD for initial, unweighted and reloaded conditions; SSC, stretch-shortening cycle; t_{flight}, flight time; $t_{contact}$, contact time; t_{stride}, stride time; $t_{braking}$, braking time; $t_{push\text{-}off}$, push-off time; $\Delta H_{braking}$ and $\Delta H_{push\text{-}off}$ for pelvic vertical displacement during the braking and push-off phases; APF, active peak force; $\bar{F}_{braking}$ and $\bar{F}_{push-off}$ for mean vertical force during the braking and push-off phases; GM, gluteus maximus; VM, vastus medialis; VL, vastus lateralis; RF, rectus femoris; STSM, semitendinosus/semimembranosus; BF, biceps femoris; SOL, soleus; GaM, gastrocnemius medialis; GaL, gastrocnemius lateralis; TA, tibialis anterior; PL, peroneus longus.

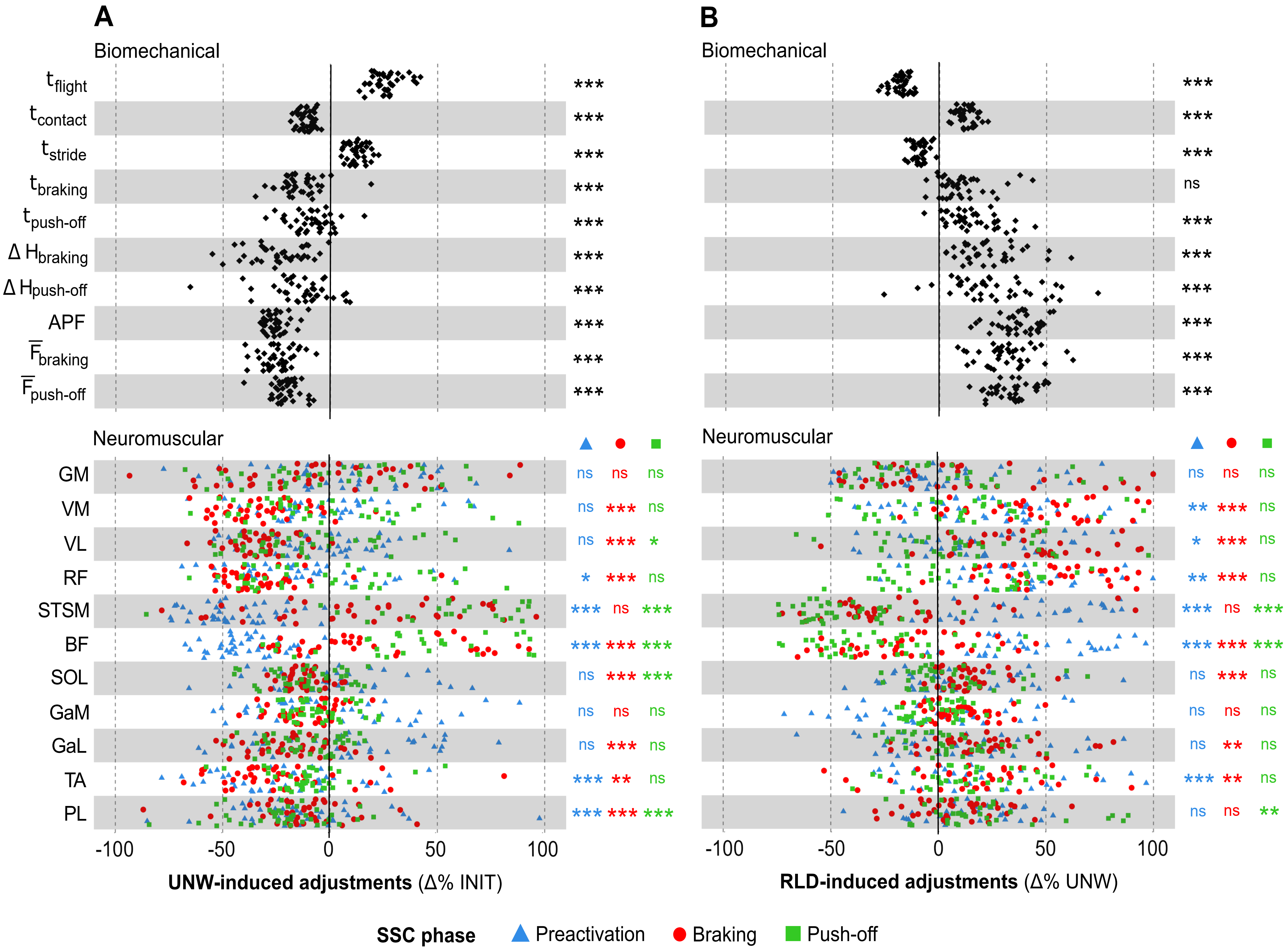
A
B
Biomechanical
t_{flight}
$t_{contact}$
t_{stride}
$t_{braking}$
$t_{push-off}$
$\Delta H_{braking}$
$\Delta H_{push-off}$
APF
$\overline{F}_{braking}$
$\overline{F}_{push-off}$
*** *** *** *** *** *** *** *** *** ***
*** *** *** ns *** *** *** *** *** ***
Neuromuscular
GM VM VL RF STSM BF SOL GaM GaL TA PL
A: GM ns ns ns; VM ns *** ns; VL ns *** *; RF * *** ns; STSM *** ns ***; BF *** *** ***; SOL ns *** ***; GaM ns ns ns; GaL ns *** ns; TA *** ** ns; PL *** *** ***
B: GM ns ns ns; VM ** *** ns; VL * *** ns; RF ** *** ns; STSM *** ns ***; BF *** *** ***; SOL ns *** ns; GaM ns ns ns; GaL ns ** ns; TA *** ** ns; PL ns ns **
-100 -50 0 50 100
UNW-induced adjustments (Δ% INIT)
RLD-induced adjustments (Δ% UNW)
SSC phase
Preactivation
Braking
Push-off
